# Artificial Intelligence-Led Whole Coronary Artery OCT Analysis; Validation and Identification of Drug Efficacy and Higher-Risk Plaques

**DOI:** 10.1161/CIRCIMAGING.125.018133

**Published:** 2025-09-25

**Authors:** Benn Jessney, Xu Chen, Sophie Gu, Yuan Huang, Martin Goddard, Adam Brown, Daniel Obaid, Michael Mahmoudi, Hector M. Garcia Garcia, Stephen P. Hoole, Lorenz Räber, Francesco Prati, Carola-Bibiane Schönlieb, Michael Roberts, Martin Bennett

**Affiliations:** Section of Cardiorespiratory Medicine, University of Cambridge, United Kingdom (B.J., X.C., S.G., Y.H., M.R., M.B.).; Department of Pathology (M.G.), Royal Papworth Hospital, Cambridge, United Kingdom.; Department of Cardiology (S.P.H.), Royal Papworth Hospital, Cambridge, United Kingdom.; Monash University, Melbourne, Australia (A.B.).; Swansea University Medical School, United Kingdom (D.O.).; Faculty of Medicine, University of Southampton, United Kingdom (H.M.G.G).; Interventional Cardiology, MedStar Washington Hospital Center, DC (M.M.).; Cardiology Department, Bern University Hospital, University of Bern, Switzerland (L.R.).; Department of Cardiology, San Giovanni Addolorata Hospital, Rome, Italy (F.P.).; Department of Applied Mathematics and Theoretical Physics, University of Cambridge, United Kingdom (C.-B.S., M.R.).

**Keywords:** artificial intelligence, biomarkers, deep learning, lipids, self-help devices

## Abstract

**BACKGROUND:**

Intracoronary optical coherence tomography (OCT) can identify changes following drug/device treatment and high-risk plaques, but analysis requires expert clinician or core laboratory interpretation, while artifacts and limited sampling markedly impair reproducibility. Assistive technologies such as artificial intelligence-based analysis may therefore aid both detailed OCT interpretation and patient management. We determined if artificial intelligence-based OCT analysis (AutoOCT) can rapidly process, optimize, and analyze OCT images, and identify plaque composition changes that predict drug success/failure and high-risk plaques.

**METHODS:**

AutoOCT deep learning artificial intelligence modules were designed to correct segmentation errors from poor-quality or artifact-containing OCT images, identify tissue/plaque composition, classify plaque types, measure multiple parameters including lumen area, lipid and calcium arcs, and fibrous cap thickness, and output segmented images and clinically useful parameters. Model development used 36 212 frames (127 whole pullbacks, 106 patients). Internal validation of tissue and plaque classification and measurements used ex vivo OCT pullbacks from autopsy arteries, while external validation for plaque stabilization and identifying high-risk plaques used core laboratory analysis of IBIS-4 (Integrated Biomarkers and Imaging Study-4) high-intensity statin (83 patients) and CLIMA (Relationship Between Coronary Plaque Morphology of Left Anterior Descending Artery and Long-Term Clinical Outcome Study; 62 patients) studies, respectively.

**RESULTS:**

AutoOCT recovered images containing common artifacts with measurements and tissue and plaque classification accuracy of 83% versus histology, equivalent to expert clinician readers. AutoOCT replicated core laboratory plaque composition changes after high-intensity statin, including reduced lesion lipid arc (13.3° versus 12.5°) and increased minimum fibrous cap thickness (18.9 µm versus 24.4 µm). AutoOCT also identified high-risk plaque features leading to patient events including minimal lumen area <3.5 mm^2^, Lipid arc >180°, and fibrous cap thickness <75 µm, similar to the CLIMA core laboratory.

**CONCLUSIONS:**

AutoOCT-based analysis of whole coronary artery OCT identifies tissue and plaque types and measures features correlating with plaque stabilization and high-risk plaques. Artificial intelligence-based OCT analysis may augment clinician or core laboratory analysis of intracoronary OCT images for trials of drug/device efficacy and identifying high-risk lesions.

**CLINICAL PERSPECTIVE**

Detailed optical coherence tomography (OCT) analysis requires time-consuming manual frame selection and measurement in specialized core laboratories and is limited by individual interpretation. Automated machine learning-based OCT analysis shows promise but can have methodological, data set, and reporting deficiencies, and many models are not sufficiently robust for clinical application. We designed and validated a modular artificial intelligence-based OCT analysis that automatically identifies plaque composition and risk. Unbiased automatic measurement of intracoronary OCT images was feasible, and could provide rapid, user-independent plaque characterization and measurements, including identification of higher-risk plaque types and changes in plaque structure associated with stabilization and reduced patient events, with a plaque classification accuracy of 83% versus histology, equivalent to expert clinician readers. Specifically, artificial intelligence-based OCT analysis replicated plaque composition changes after statin use, including reduced lipid arc and increased fibrous cap thickness_min_, and could identify high-risk plaque features leading to major adverse cardiovascular events including minimal lumen area <3.5 mm^2^, Lipid arc >180°, and fibrous cap thickness <75 µm, similar to a core laboratory. Although prospective studies are required, artificial intelligence-based assistive technology may be used for rapid and comprehensive assessment of patients before percutaneous revascularization or identification of drugs or devices that are likely to be successful in Phase 3 trials. This technology may allow reliable prognostic stratification that would improve management of patients with coronary artery disease, for example, by adopting preventive strategies and approaches aimed at early diagnosis and treatment of high-risk atherosclerosis.

Intracoronary optical coherence tomography (OCT) can identify high-risk plaques and is a widely used surrogate efficacy marker for drug/device studies. For example, fibrous cap thickness (FCT) <75 µm, minimum lumen area <3.5mm^2^, lipid arc >180°, and presence of macrophages, calcific nodules, neovascularization, and cholesterol crystals^1–6^ are associated with major adverse coronary events (MACE). Many of these features change with drug/device therapy, including drugs that reduce patient events with minimal changes in plaque volume,^7–13^ suggesting plaque stabilization.

However, real-world OCT pullbacks are rich data sets containing hundreds of images and tens-of-thousands of candidate measurements/artery. Consequently, detailed OCT analysis for clinical trials currently requires time-consuming offline manual frame selection and measurement in specialized core laboratories. Furthermore, inter- and intraobserver variability for particular tissues, measurements, and plaque types is suboptimal,^14–16^ and even between core laboratories.^17^ Such variability may contribute to the low event rates observed for high-risk OCT features (<3% individually and 3.7% in combination^5^) with positive predictive values of only 20% to 30%,^18–20^ and limit the ability of OCT to identify high-risk plaques in real-time. A fully automated, time-efficient OCT analysis system could improve OCT reproducibility, and several systems have been described.^21–26^ However, many systems are still limited by the high frequency of artifacts and the similarity of artifact to disease,^27^ and their validation, generalizability, and accuracy on whole OCT pullbacks in real-world clinical scenarios are unclear.^28^ For example, many models used small or highly selected training data sets (eg, excluding frames containing stents or artifacts) and frequently lack either histopathologic validation or external validation against core laboratories using large-scale clinical trial data to substantiate model performance.

We designed and tested artificial intelligence-based OCT analysis (AutoOCT), a deep learning artificial intelligence (AI)-based intracoronary OCT analysis system to overcome these limitations and provide rapid, fully automatic, user-independent plaque characterization and measurements. AutoOCT could be a very valuable tool for trials of antiatherosclerosis drugs and identification of higher-risk plaques.

## Methods

### Study Population

Because of the sensitive nature of the data collected, data access requests from qualified researchers trained in human subject confidentiality protocols may be sent to the senior author. The postmortem study was approved by the Cambridgeshire 3 Research Ethics Committee (07/H0306/123) and all relatives provided informed consent. The IBIS-4 (Integrated Biomarkers and Imaging Study-4) and CLIMA (Relationship Between Coronary Plaque Morphology of Left Anterior Descending Artery and Long-Term Clinical Outcome Study) studies were approved by the institutional review boards of all participating centers and all patients provided written informed consent.

To develop AutoOCT, we annotated 36 212 OCT frames from 127 complete OCT pullbacks from 106 unselected patients with coronary artery disease from 3 UK cardiothoracic centers. All patients provided informed consent, and all pullbacks were included for analysis with no exclusion criteria. Internal histopathologic validation used a coregistered OCT/histology data set from 13 postmortem patients with written consent from relatives.^29^ External validation used all 83 patients from the Integrated Biomarker Imaging Study-4 OCT arm (IBIS-4, REGISTRATION: URL: https://www.clinicaltrials.gov; Unique identifier: NCT00962416)^7^ and 31 MACE and 31 control patients from the CLIMA study (NCT02883088).^5^ All pullbacks were acquired with frequency-domain C7-XR or OPTIS systems (Abbott Vascular, Santa Clara, CA) using a nonocclusive technique.

### Deep Learning Model Development and Training

AutoOCT was developed in Python (3.8) with training facilitated using the Cambridge University high performance computing cluster. Segmentation masks for different artery structures and plaque components were extracted using a DeepLabv3+ convolutional neural network architecture. The model was trained with annotated frames in axial cross-sections after grayscale conversion, with a (512, 512) spatial size. Data were split at a patient level ensuring that OCT images could not be utilized in both testing and validation, and consecutive frames could not be used in both training and validation sets. Data were randomly divided into training, testing and validation sets in a 14:1:1 patient-level ratio, respectively, strictly avoiding data repetition. Hybrid Dice (similarity between 2 segmentations, ranging from 0 to 1, where 1 indicates 2 segmentations are identical, and 0 indicating no overlap) and cross entropy loss were utilized for training with adaptive moment estimation as the optimizer. A custom-designed data loader was used to overcome class imbalances and data preprocessed and optimized to remove artifacts before use in training. Extensive ablation studies with the validation data set (n=10 pullbacks, 10 patients) aided best model architecture selection. For plaque classification, an additional AI-based module utilizing an EfficientNet architecture was developed from 14 028 IBIS-4 OCT frames (83 patients) and divided with a patient-level stratification into training (7904 frames, 52 patients), validation (2878 frames, 14 patients), and testing (3246 frames, 17 patients) sets. Images were converted to polar orientation around the lumen center and resized to a spatial resolution of (512, 512) before training with a comprehensive augmentation pipeline to enhance model generalization, including random horizontal rolling, flips, color jittering, and grayscale conversion. For classification, we utilized a 2-step system first categorizing vessel segments as: (1) low risk (normal vessel, adaptive intimal thickening, pathological intimal thickening), or (2) higher-risk (fibrocalcific plaque, thick cap fibroatheroma [ThCFA], and thin cap fibroatheroma [TCFA]), with more detailed classification of higher-risk plaques based on measurement of plaque components (eg, FCT_min_). The adaptive moment estimation optimizer was used for training, a learning rate-scheduler to mitigate over-fitting, and a custom imbalanced data set sampler to address class imbalance.

### Data Annotation and Plaque Definitions

OCT pullbacks were exported in DICOM (Digital Imaging and Communications in Medicine) format for offline analysis. Manual segmentation of frames was performed using the Medical Imaging Interaction Toolkit (v2021.10) software. All ground-truth annotation was performed in axial cross-sections by an experienced intravascular imaging specialist following accepted plaque definitions.^6^ All frames were labeled, regardless of classification, data quality, or presence of imaging artifacts, but excluding frames within guide catheters or stents which were noted using binary labels. Lumen contours, and the external elastic lamina were defined, with structures in-between classified as guidewire shadow, bifurcations, or as plaque components. Normal vessel and fibrous tissue were annotated as 1 structure, but each plaque component was annotated separately. AutoOCT definitions of different plaque morphologies are described in the Supplemental Material.

### Measurements and Statistical Analysis

Continuous variables are summarized as median (interquartile range) or were stated as mean±SD and categorical variables as counts (percentage). Agreement between measurements (manual or AutoOCT) or with histology measurements was compared using intraclass correlation coefficients (ICCs) for absolute agreement and Bland-Altman plots comparing mean against difference in measurements. Plaque classification accuracy was assessed by diagnostic performance with Wald-type asymptotic tests of noninferiority. *P* values were reported for exploratory purposes for model performance against clinical studies, without any claims of significance. Paired Student *t*-, Wilcoxon signed-rank-, and χ^2^ tests were applied when appropriate. Two-sided *P* values are reported throughout adopting 0.05 as the significance threshold, excepting Wald-type asymptotic tests of noninferiority^30^ with continuity correction for sample size for which 0.025 was adopted. The noninferiority margin was set at 0.1. Analyses were performed using SPSS 28.0.0 (SPSS, Inc, IBM Computing).

To ensure robustness and reproducibility of the methods described, AutoOCT and this article were scored against the Checklist for Artificial Intelligence in Medical Imaging^31^ and the Consensus-based Recommendations for Machine-learning-based Science^32^ checklists. Both are provided in the Supplemental Material.

## Results

### Study Population Characteristics

**Figure 1.**
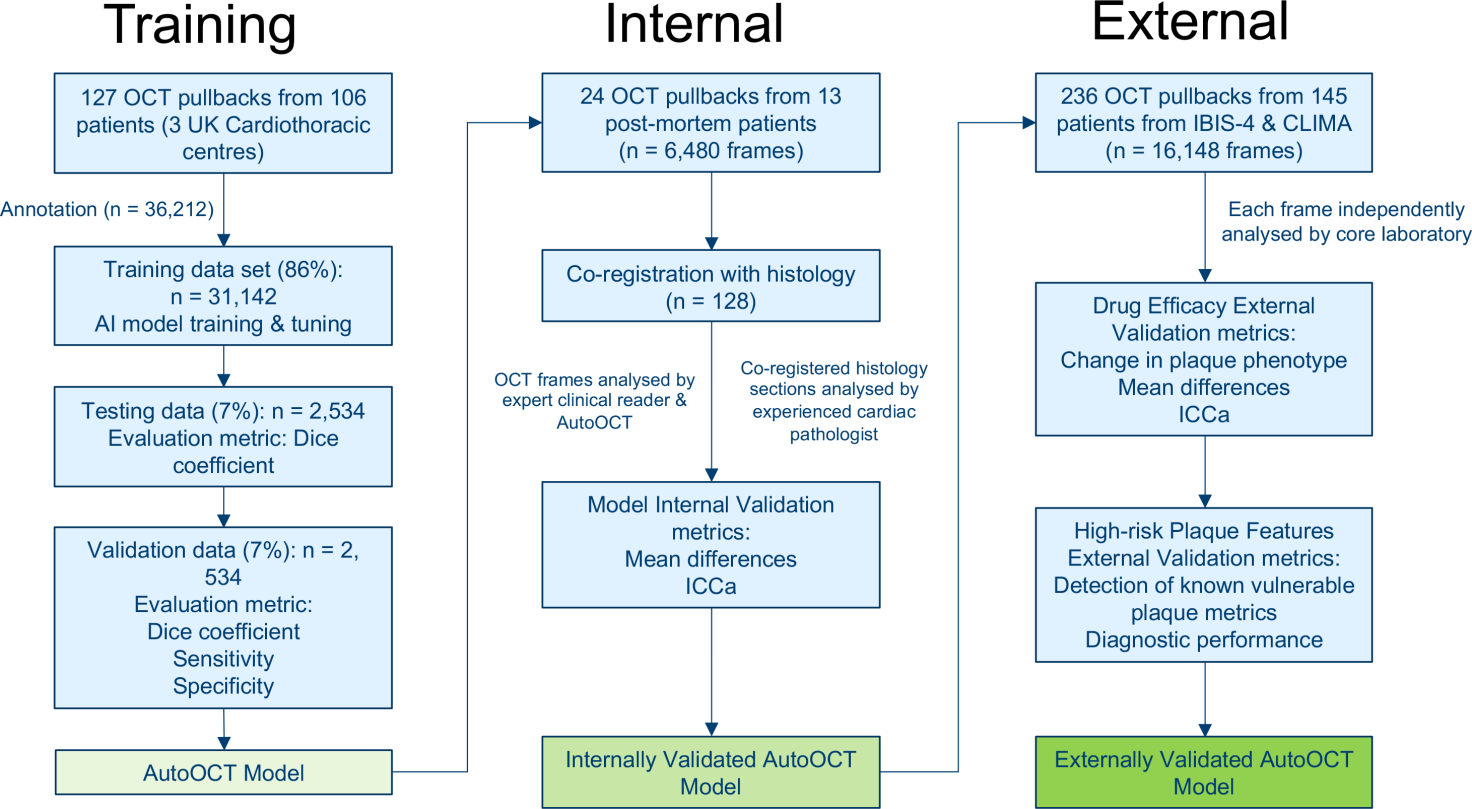
**Study structure: training, internal, and external validation.** Artificial intelligence-based OCT analysis (AutoOCT) was trained with annotated optical coherence tomography (OCT) frames from 127 complete pullbacks from unselected patients from 3 UK cardiothoracic centers. The model was tested on a holdout group of 2534 frames, and AutoOCT performance internally validated on OCT frames from ex vivo OCT pullbacks co-registered with histology from postmortem arteries. AutoOCT performance was externally validated by comparison with findings from 2 large clinical trials, the IBIS-4 (Integrated Biomarker Imaging Study-4) trial that examined 13 m treatment with high dose rosuvastatin, and the CLIMA (Relationship Between Coronary Plaque Morphology of Left Anterior Descending Artery and Long Term Clinical Outcome Study) study that identified higher-risk plaque features leading to major adverse cardiovascular events (MACE).

Overall, 366 pullbacks from 297 patients were analyzed, representing 58 840 OCT frames. Separate data sets were used for training (106 patients, 127 whole pullbacks, 36 212 frames from unselected patients from 3 UK centers), histopathologic validation (13 patients, 24 whole pullbacks, 6480 frames), and external validation (145 patients, 236 pullbacks, 16 148 frames) from IBIS-4 (83 patients) and CLIMA (62 patients) studies (Figure 1). Autopsy donors were aged 47 to 85 years, 71.4% male, and died from cardiovascular or noncardiovascular causes (Table S1). Full IBIS-4 and CLIMA patient characteristics are described in their respective publications.^5,7^

### Model Performance

**Figure 2.**
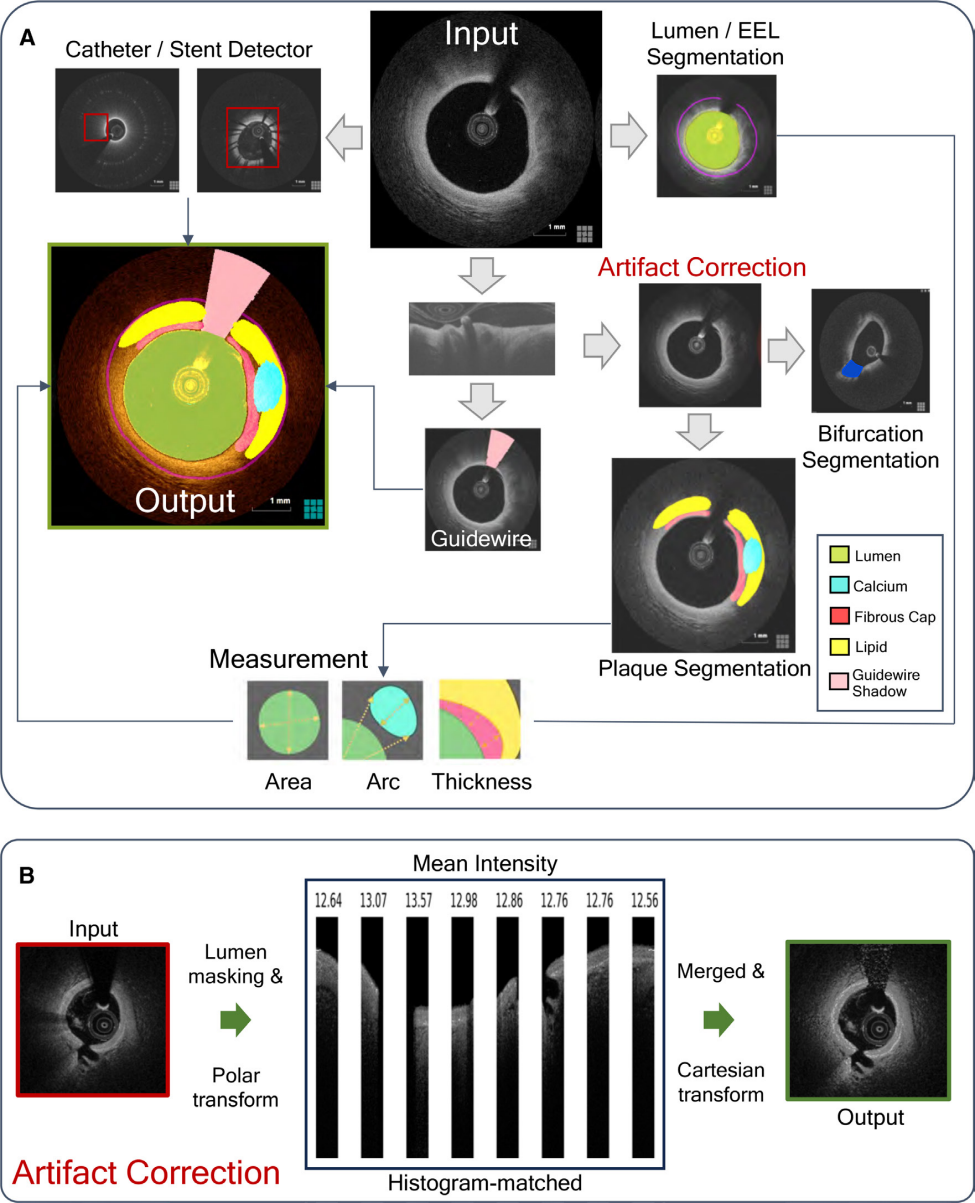
**Deep learning model outline and artifact correction. A**, Input axial optical coherence tomography (OCT) frames were preprocessed before undergoing parallel processing. Catheter and stent locations were first classified with frame locations outputted. Plaque components were then segmented in either polar transform or Cartesian images. **B**, Before plaque component segmentation, images underwent correction of the effects of artifacts on the vessel wall using masking, polar transform, histogram matching, and retransform. Measurements were made of segmented plaque components with outputted data comprising clinically useful measurements and reconstructed, labeled OCT-images.

**Figure 3.**
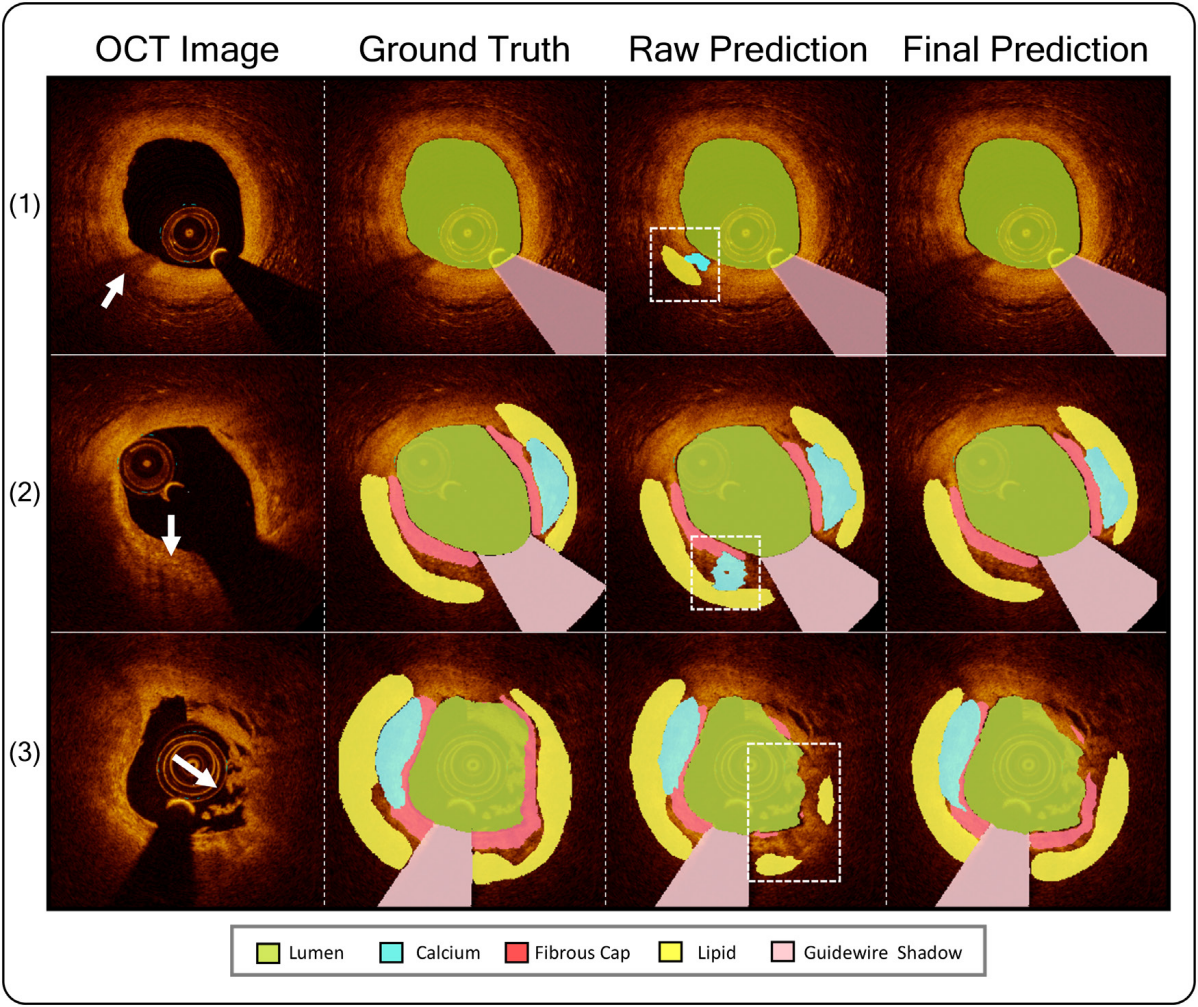
**Plaque segmentation before and after artifact correction.** Examples of plaque segmentation in frames containing optical coherence tomography (OCT) artifacts. From **left** to **right**, the raw OCT image, ground truth/manual annotations, artificial intelligence-based OCT analysis (AutoOCT) raw prediction before optimization, and final prediction after optimization are shown, respectively. (1) Gas bubble artifact; (2) Macrophage dots causing signal drop-out; (3) Plaque rupture with resulting signal drop-out. Arrows denote artifacts, outlined areas denote segmentation errors.

AutoOCT was designed with sequential modules to detect the guide catheter or stents, segment artery or imaging components (lumen, external elastic lamina, guidewire shadow), correct the effect of common artifacts on the underlying vessel wall, and then segment and measure individual plaque components (Figure 2A). AutoOCT performed very well on testing data for whole pullback artery and imaging components (Dice: Lumen 0.99, external elastic lamina 0.99, Guidewire shadow 0.96), and both guide catheter and stents (Table S2), indicating that segmentation and recognition of these components closely replicated ground truth. A novel optimization technique based on histogram matching (Figure 2B and Supplemental Material) was used to remove the effects of artifacts on the underlying vessel wall and improve segmentation accuracy in complex plaque morphologies such as rupture (Figure 3). Good performance (Dice 0.8 or above) was subsequently achieved for whole-pullback plaque composition on testing data (Dice: Lipid 0.84, Calcium 0.85, Fibrous cap 0.80; Table S2) and measurements correlated excellently (ICCa 0.7 or above) with ground-truth (lipid arc ICCa, 0.94 [95% CI, 0.91–0.97], calcium arc 0.88 [95% CI, 0.79–0.95]). Consistency of plaque classification performance was undertaken, checking between the internal validation, internal testing, and external holdout cohorts^33^ to identify drift in model predictions and optimal operating points (Figure S1). AutoOCT analysis of a full pullback comprising 271 to 540 axial frames of 512×512 pixels took ≈180 to 300s.

### AutoOCT Validation Against Histopathology and Expert Reader

AutoOCT was next validated using ex vivo OCT pullbacks obtained under physiological pressures with matched histopathology.^29^ 128 unique OCT frames were co-registered with corresponding histological sections and lesions classified histologically by an expert cardiovascular pathologist (M.G.) as normal vessel (n=3, 2.3%), adaptive intimal thickening (n=19, 14.8%), pathological intimal thickening (n=29, 22.7%), fibrocalcific (n=8, 6.3%), and fibroatheroma (n=69, 53.9%). 22 (17.2%) fibroatheromas were TCFA (FCT<75 µm, median FCT 58.3 µm [50.0–65.8]) and 47 were ThCFA (median FCT 113.3 µm [85.0–140.0]).

**Figure 4.**
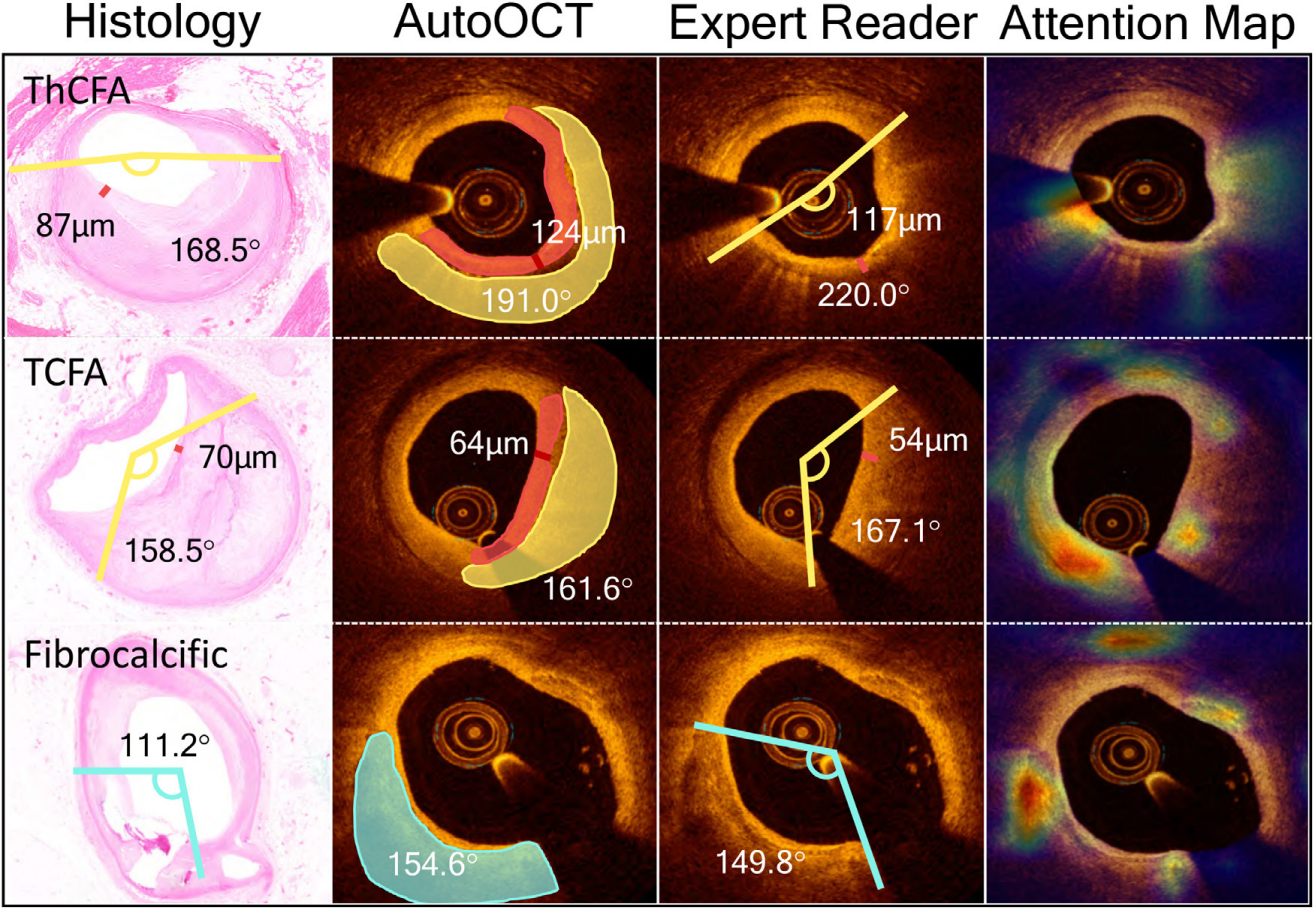
**Artificial intelligence-based OCT analysis (AutoOCT) performance in high-risk lesions. Left** to **right**, Plaque components for higher-risk plaque-types measured on histology sections, coregistered optical coherence tomography (OCT) frames by AutoOCT, and expert OCT reader, and attention map output from the plaque classification system. Lipid and calcium arcs are labeled in yellow and blue respectively as degrees, and fibrous cap thickness (FCT) by red line in microns. TCFA indicates thin cap fibroatheroma; and ThCFA, thick cap fibroatheroma.

We compared plaque component measurements from AutoOCT with histology and an expert interventional cardiologist reader (SH), focusing on higher-risk lesions (Table 1). Overall AutoOCT measurements showed excellent correlation with expert reader (ICCa, 0.86 [95% CI, 0.84–0.88]; *P*<0.001). AutoOCT-derived measurements were generally similar to histology, and particularly for lipid arc (181.8° (122.9–248.7) versus 156.9° (118.4–222.8), *P*=0.945) and in AutoOCT-defined TCFA (Minimum FCT [FCT_min_] 48.0 µm [40.0–60.3] versus 58.3 µm [50.0–65.8], *P*=0.674; Table 1; Figure 4). Expert-defined FCT_min_ was slightly higher than histology in ThCFAs (145.0 [99.0–233.0] versus 113.3 [85.0–140.0]) but not in TCFAs (50.0 [32.3–52.5] versus 58.3 [50.0–65.8]), and for combined TCFAs and ThCFAs. FCT_min_ was similar for AutoOCT versus histology: *P*=0.451, expert versus histology: *P*=0.417, or AutoOCT versus expert: *P*=0.757). AutoOCT was able to identify histologically defined lower- (normal vessel, adaptive intimal thickening, pathological intimal thickening) or higher-risk (Fibrocalcific, ThCFA, TCFA) plaque-types with a similar accuracy to an expert OCT reader (83% versus 84%). Refining higher-risk plaque classification using plaque component measurements (Supplemental Material), demonstrated overall diagnostic accuracy of AutoOCT of 70% to 91% for different lesions, and 78.1% for TCFA, and noninferior to an expert OCT reader (*P*<0.025 for all plaque-types; Table 2).

**Table 1.**
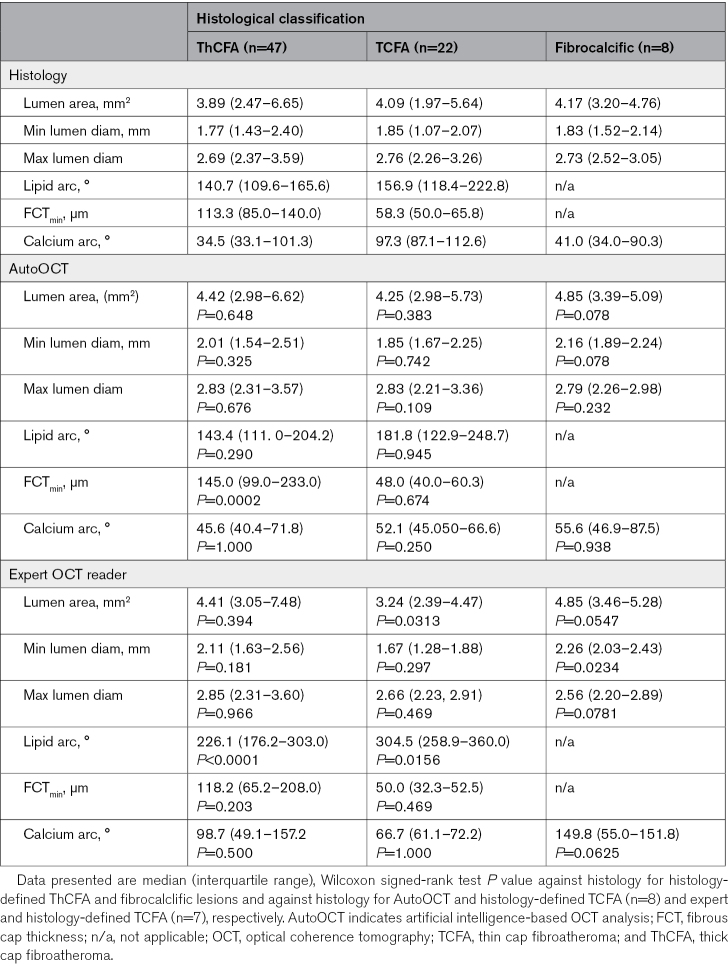
Histological, AutoOCT, and Expert Reader OCT Features for Each Plaque Subtype

**Table 2.**
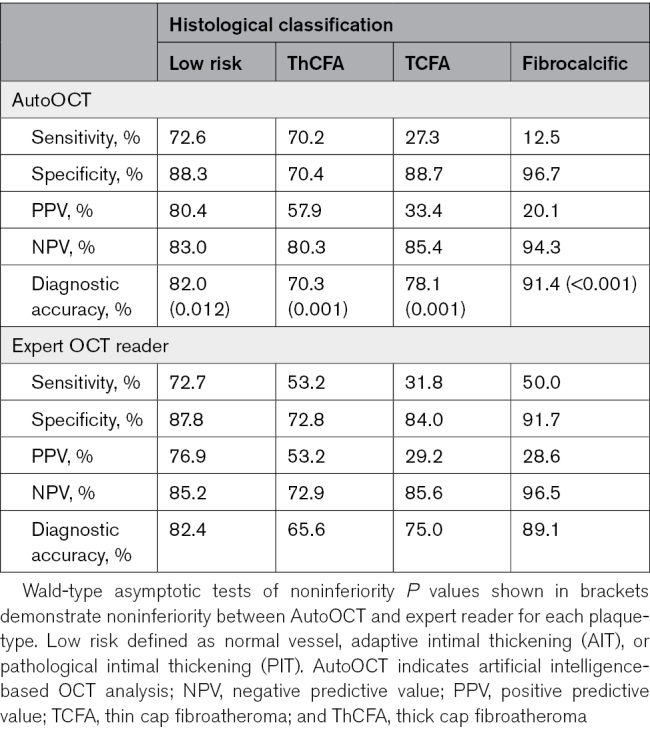
Accuracy of AutoOCT and Expert Reader Plaque Classification Compared With Histology

### AutoOCT External Validation Against Core Laboratory: Drug Efficacy

**Figure 5.**
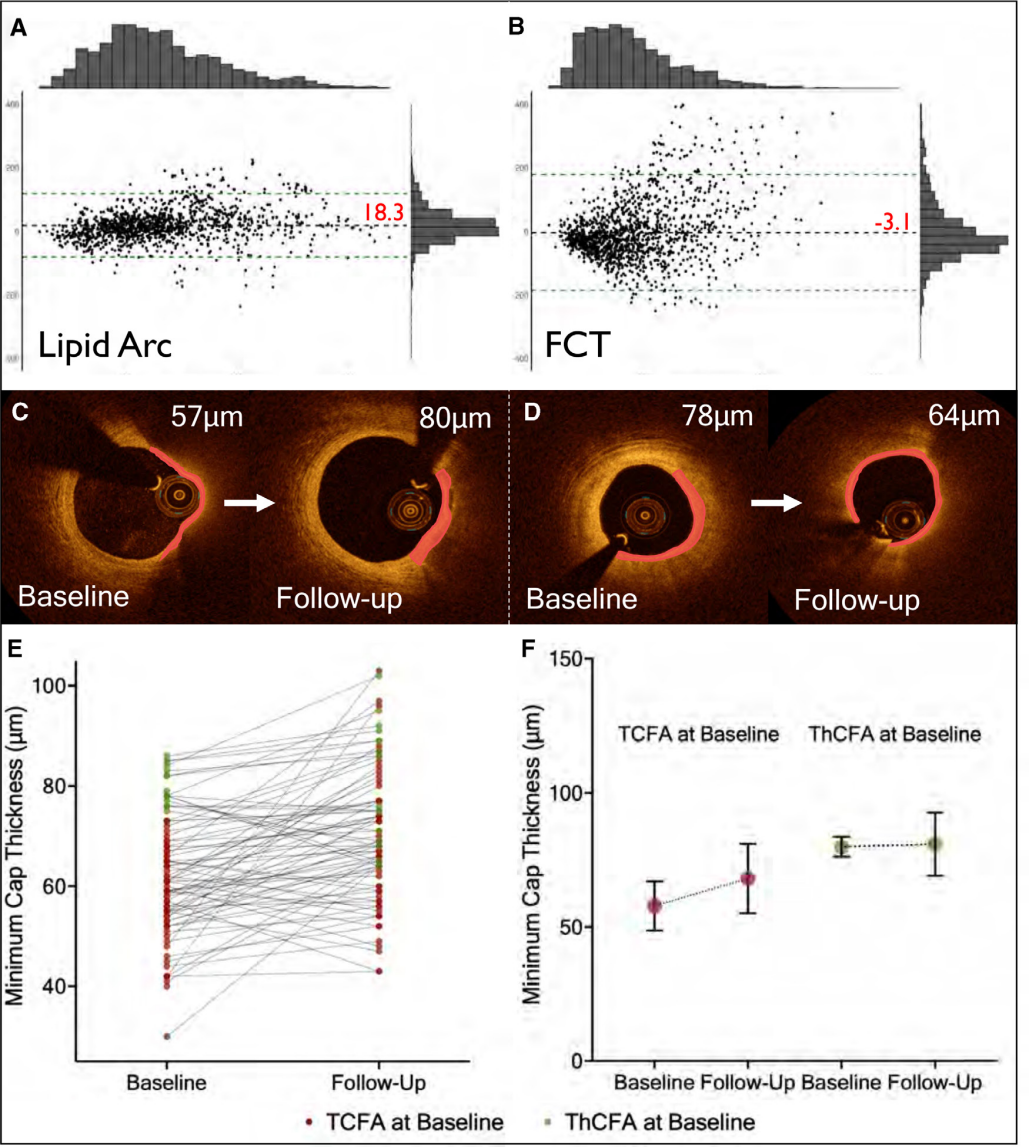
**Artificial intelligence-based OCT analysis (AutoOCT) validation of drug effects against core laboratory. A** and **B**, Bland-Altman plots of mean (*x* axis) and difference (*y* axis) with histograms of mean (**top**) and difference (**right**) for measurements of lipid arc (n=1218; **A**) and minimum fibrous cap thickness (FCT; n=1384; **B**). **C** and **D**, Example fibroatheroma lesions that show regression of thin cap fibroatheroma (TCFA; **C**) or progression of thick cap fibroatheroma (ThCFA; **D**) after statin therapy. Fibrous caps are outlined in red. **E** and **F**, Graphs for minimum FCT at baseline and follow-up for individual fibroatheromas (**E**) or mean FCT (**F**) for TCFA and ThCFA (n=31).

Although AutoOCT performed well on selected images matched with histology, AI-based OCT studies in clinically relevant scenarios and real trial data are limited. We therefore undertook external validation against external core laboratories using frame-based comparison from 2 clinical trials. The IBIS-4 OCT substudy showed that 13 m of high-intensity statin treatment increased FCT_min_, reduced lipid arc, and 5.8% of lesions and 69.2% TCFA regressed to more stable plaque phenotypes. Of all 83 patients (153 arteries), 27 patients (31 arteries) had ThCFA or TCFA at both time points. AutoOCT lipid arc measurements demonstrated good correlation with core laboratory measurements (ICCa, 0.75 [95% CI, 0.68–0.80]; *P*<0.001) with clinically acceptable average differences 18.3±58.8° (*P*<0.001) and 93.6% (1140/1218) measurements within 95% CI (Figure 4A). AutoOCT FCT_min_ also correlated well with core laboratory measurements (ICCa, 0.66 [95% CI, 0.62–0.70]; *P*<0.001), with a nonsignificant and subpixel-level average difference (3.1±94.6 µm [*P*=0.241]), and 93.7% (1297/1384) measurements within 95% CI (Figure 4B). Both whole-vessel AutoOCT FCT_min_ and lipid arc showed a similar increase or decrease respectively to core laboratory analysis (FCT 62.9±28.4 µm to 81.8±33.4 µm, *P*<0.001 versus 64.88±19.89 µm to 87.88±38.08 µm, *P*=0.008; lipid arc 63.1±21.7° to 49.8±20.3°, *P*<0.001 versus 55.94±31.04° to 43.46±3.48°, *P*=0.013; Figure 5C and 5D; Table S3).

IBIS-4 also reported changes in plaque types with drug treatment, so we compared AutoOCT-based classification against core laboratory definitions. Changes in AutoOCT lesion mean FCT_min_ was similar to the core laboratory (76.7±36.1 µm to 83.0±35.3 µm versus 74.0±32.3 µm to 94.2±39.9 µm), mostly driven by TCFA (Figure 5E). AutoOCT increased FCT_min_ occurred in 82.0% TCFA (92.3% by core laboratory) compared with 58.3% of ThCFA (52.2% by core laboratory; Figure 5F), suggesting that high-intensity statin treatment increases FCT_min_ mostly in TCFA.

### AutoOCT External Validation Against Core Laboratory: High-Risk Plaque Features

**Figure 6.**
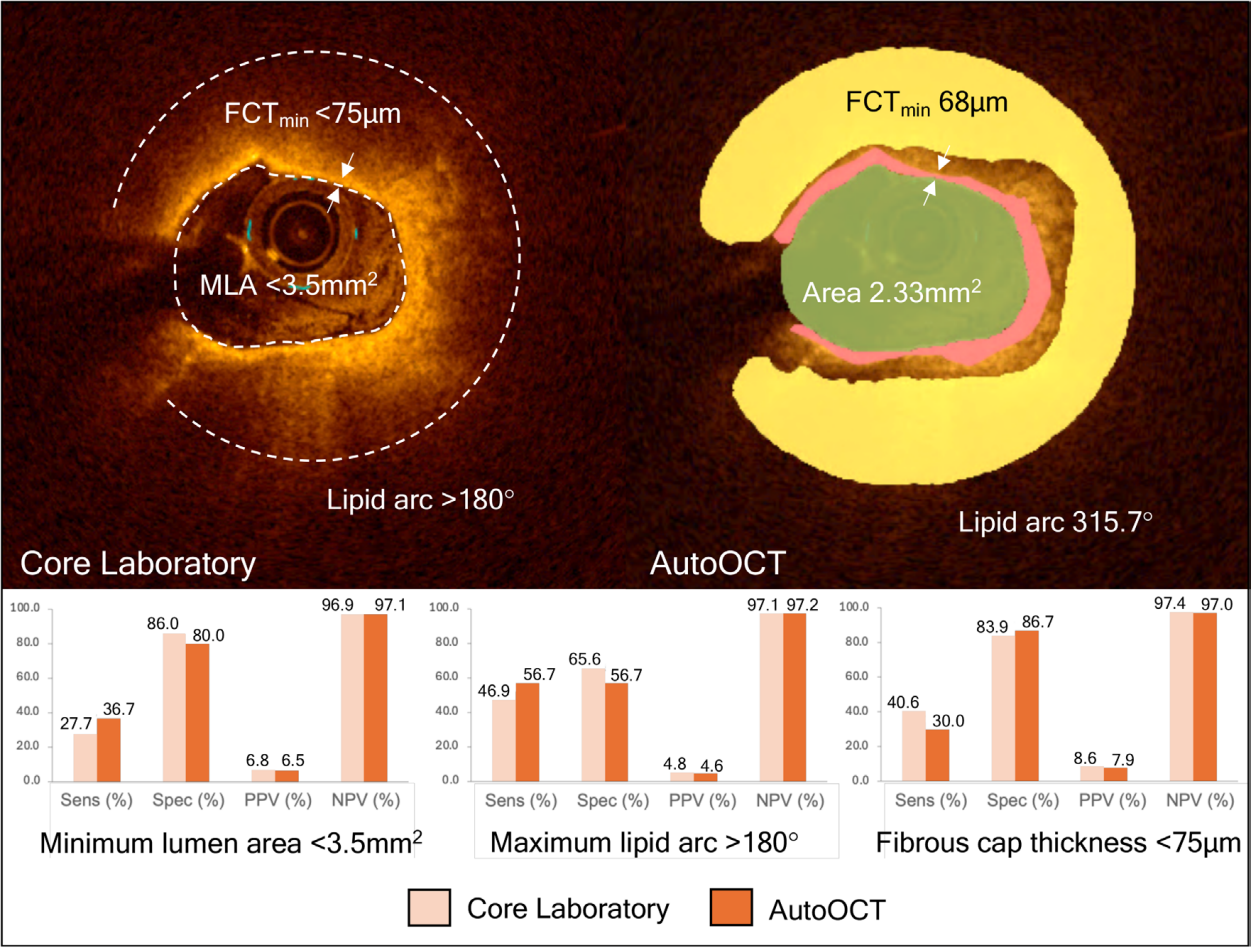
**Validation of artificial intelligence-based OCT analysis (AutoOCT) to detect higher-risk plaque features against core laboratory. Upper**: Vulnerable plaque features determined by core laboratory (**left**) and AutoOCT (**right**). **Lower**: AutoOCT diagnostic performance for each plaque characteristic compared with the core laboratory. FCT indicates fibrous cap thickness; MLA, minimum lumen area; NPV, negative predictive value; and PPV, positive predictive value.

The CLIMA study^5^ of untreated proximal left anterior descending arteries showed that minimum lumen area <3.5 mm^2^, FCT <75 µm, and lipid arc >180° on OCT were associated with 1-year MACE (composite end point of cardiac death and target segment myocardial infarction. We studied 62 participants (31 MACE and 31 controls), with similar patient and lesion features (Supplemental Material, Table S4). AutoOCT showed that more MACE patients had minimum lumen area <3.5 mm^2^ (38.7% versus 19.4%; *P*<0.001), FCT <75 µm (29.0% versus 12.9%; *P*<0.001), and maximum lipid arc >180° (54.8% versus 41.9%; *P*<0.001), similar to core laboratory analysis of our subset (Table S5). Although the sensitivity and specificity of different OCT criteria to predict MACE varied, AutoOCT and core laboratory positive predictive value, negative predictive value, and diagnostic accuracy of each variable were similar (Figure 6 and Table S6), suggesting that AutoOCT can identify features of plaque vulnerability.

## Discussion

We designed and tested a modular deep learning AI-based image analysis system for intracoronary OCT, including correction of segmentation errors induced by common artifacts and both internal and external validation to detect and measure multiple markers of disease progression/regression and higher-risk plaques. Importantly, AutoOCT was trained using whole pullbacks from unselected patients, representative of real-world clinical practice, and not only perfect, artifact-free images with classical architecture features and known measurements. Our key findings are (1) AutoOCT could recover images containing common artifacts; (2) AutoOCT-derived plaque classification correlated well with histology; (3) AutoOCT-derived identification and measurement of higher-risk features such as FCT and lipid arc were comparable to histopathology, correlated well with an expert reader, and accurately identified TCFA; (4) AutoOCT replicated core laboratory findings consistent with plaque stabilization after high-intensity statins and features of plaque vulnerability that predict MACE, including minimum lumen area <3.5 mm^2^, FCT <75 µm, and lipid arc >180°.

Despite reported success of deep-learning models for intracoronary OCT imaging, many models are trained and tested with small, curated data sets with limited disease diversity and highly selected frames that exclude common artifacts from stents, poor image quality, thrombus, plaque rupture, dissection, and bifurcations that may not represent real-world algorithm performance.^26^ In contrast, AutoOCT was trained with whole unselected pullbacks (average 285 frames/patient), which is crucial for generalizability and real-world application, and used preprocessing to mitigate effects of artifacts, optimize poor-quality images, and allow analysis of all available data. Many studies report identification of lumen or individual plaque components rather than the overall plaque phenotype through collating multiple features.^26,28^ In contrast, AutoOCT derives binary segmentations for tissues followed by a measurement pipeline which combines labels, allowing multiple tissue types to be identified and measured to identify lower versus higher-risk plaques (adaptive intimal thickening and pathological intimal thickening versus TCFA, ThCFA, and fibrocalcific). Finally, many studies lack validation against histopathology, and most lack validation against core laboratory analyses of individual frames. We used a well-curated database of real-world clinical OCT pullbacks from 3 centers for training, a separate data set for internal validation, and externally validated AutoOCT against core laboratory analysis of 2 large-scale landmark clinical trials. While improvements continue, the current algorithm replicated core laboratory performance.

Our study demonstrates that AI-based OCT analysis may aid drug and device development, and trial design and analysis for natural history studies. For example, increased FCT, reduced lipid arc, TCFA regression, and reduced ThCFA progression can represent a signature of a drug/device likely to reduce MACE. AutoOCT of coregistered baseline and follow-up images showed accurate and reproducible vessel and frame-based analysis of these features, and gave similar results to the IBIS-4 study core laboratory. AutoOCT may therefore allow fast, automated identification of features of drug efficacy and changes in plaque morphology in small numbers of patients over short time-frames.

Histopathology and multiple imaging studies have identified the substrate underlying many MACE.^1,5,34–38^ However, studies to identify features predictive of MACE show a high prevalence of vulnerable plaque features but low positive predictive values^18–20^ and require large patient numbers often studied for 3 to 5 years. Furthermore, study analysis is labor-intensive, time-consuming, and requires expert interpretation. AutoOCT had a frame-level accuracy to detect TCFA of 78.1% ex vivo and FCT negative predictive value of >97% for MACE in vivo. While AutoOCT positive predictive value for MACE was low and similar to core laboratory analysis in vivo, AutoOCT positive predictive value for detecting TCFA ex vivo was 33.4%, representing noninferior performance compared with an expert-reader. While AI-based OCT analysis may not replace core laboratories, whole vessel and frame-based analysis in minutes/pullback may greatly speed up the analysis process.

### Limitations

Our study has some limitations. We used all frames for training, regardless of patient characteristics, image quality, presence of artifacts, or plaque phenotype. Having no exclusion criteria increases sensitivity, but reduces specificity to detect plaque components. In addition, all OCT data utilized was generated using Abbott systems and formal validation of our findings on other manufacturers’ images is ongoing. Importantly, our model was trained with data representative of real-world clinical practice, and all data utilized has been provided by researchers undertaking independent studies or clinical work. In addition, all data was used to validate AutoOCT to avoid selection bias and create an OCT system trained and tested representative of real-world OCT data. We have also demonstrated generalizability on 3 independent data sets, and showed similar measurements to core laboratories, with metrics within the published variability for OCT analysis.^15,16,39,40^ Second, our postmortem study examined frames from 13 OCT pullbacks and findings should be validated in larger data sets; however, 128 OCT frames with 128 ROI were coregistered with histology from the entire pullback rather than just specific plaque types, suggesting a robust applicability to clinical OCT. In addition, the validation of OCT measurements against histopathology is limited by very different resolutions, nonuniform shrinkage, and tissue destruction, particularly affecting measurements such as FCT_min_ of small structures, although every effort was made to mitigate modality differences through perfusion fixation of specimens. Coregistration between OCT and histology is also challenging, and small longitudinal mismatches may also influence correlations. However, an experienced imaging specialist (B.J.) performed all coregistration blinded to plaque classification. Similarly, our study utilized a single expert OCT reader and expert cardiovascular pathologist. However, all analysis was performed blinded and while other groups have utilized multiple readers or pathologists, even specialized core laboratories with multiple readers differ in opinion both internally and with other core laboratories.^14–17^ Third, although preprocessing can correct segmentations due to most imaging artifacts, it cannot restore some artifacts that mimic TCFA including tangential signal dropout.^27^ However, we report similar positive predictive value and overall diagnostic accuracy for TCFA compared with an expert reader. Fourthly, several analysis errors are due to technical limitations of OCT, such as light shielding in dense fibrocalcific plaque and similarity between tissues. These issues may explain both differences between AutoOCT and expert analysis for lipid and FCT, but are shared by both human and AI-based systems (Figure S2). Fifth, while the current version of AutoOCT can identify stent location, length and size, at present the model is not trained to measure features such as edge dissection and malposition. Finally, although our subset of CLIMA patients was similar to the entire cohort, the absolute prognostic value of each AutoOCT-defined parameter will require analysis of the whole 1003 patients over full follow-up. In addition, although both IBIS-4 and CLIMA are prospective studies comparing baseline imaging and outcomes, our analysis was retrospective and further prospective studies utilizing prespecified AutoOCT-defined higher-risk features at baseline would be informative.

### Conclusions

We developed and validated a highly generalizable deep learning AI-based model utilizing real-world clinical data for automatic coronary OCT plaque characterization. Our model utilized image preprocessing to correct segmentation errors and optimize poor-quality OCT images containing artifacts and may thus reduce subjectivity and increase reproducibility in image interpretation. AutoOCT demonstrated the small changes in plaque composition seen with pharmacotherapy and identified features of plaque vulnerability, illustrating its potential in research and real-time plaque classification and identification of higher-risk lesions to inform patient management.

## Article Information

### Sources of Funding

Supported by British Heart Foundation Grants PG/18/14/33562, RG13/14/30314, RE/24/130011, TA/F/20/210001 (London), Academy of Medical Sciences Starter Grants for Clinical Lecturers (REF: SGL030\1012), Innovate UK Advancing Precision Medicine 10069871, National Institutes of Health, R01 HL150608, EPSRC Cambridge Maths in Healthcare (Nr. EP/N014588/1) and Cambridge NIHR Biomedical Research Centres.

### Disclosures

Dr Hoole has been an advisor to Abbott Vascular and holds share options in Octiocor. Dr Räber received research grants to the institution by Abbott, Biotronik, Boston Scientific, Infraredx, Sanofi, Regeneron and consultation/speaker fees by Abbott, Biotronik, Gentuity, Medtronic, Novo Nordisk, and Occlutec. Dr Prati is a consultant for Abbott, Amgen, and Novo Nordisk; and has received speaker fees/honoraria from Sanofi. Drs. Roberts and Bennett are founders of Octiocor, Ltd. The other authors report no conflicts.

### Supplemental Material

Supplemental Methods

Figures S1–S2

Tables S1–S6

Reference 41

## Supplementary Material


