# Supplementary material for: Artificial Intelligence-Led Whole Coronary Artery OCT Analysis; Validation and Identification of Drug Efficacy and Higher-Risk Plaques
Source: Circ Cardiovasc Imaging. 2025 Sep 25;18(11):e018133. doi: 10.1161/CIRCIMAGING.125.018133 (PMC12622268; doi:10.1161/CIRCIMAGING.125.018133)
Supplement: Supplementary file 1 [file hci-18-e018133-s001.pdf]

# Supplemental Material

# Supplemental Methods

### Histopathological and clinical validation of AutoOCT

Left anterior descending arteries from 13 donors underwent OCT imaging post-mortem before co-registration with histopathology as described previously[29]. This dataset comprised 1290 mm of coronary artery with 207 histological regions of interest (ROI) co-registered with OCT, and we used 128 OCT frames with 128 ROI to validate AutoOCT. OCT pullbacks were analyzed for plaque classification and lumen, EEL, and plaque parameter measurements by AutoOCT and an expert interventional cardiologist with >10 years' experience (total intravascular imaging experience: >500 IVUS and >500 OCT procedures), blinded to AutoOCT results. Histological plaque classification and measurements were validated by an independent experienced cardiac pathologist, blinded to coronary imaging and AutoOCT.

### Histological processing and co-registration

After imaging, arteries were perfusion-fixed in 10% buffered formalin for ≥24 hours. 5μm sections were cut at 400μm intervals, maintaining proximal and distal orientation. Sections were stained with hematoxylin-eosin and van Gieson. OCT images were matched to co-registered histological sections by an experienced intravascular imaging investigator, blinded to final histological plaque classification. Detailed measurements were taken during *ex-vivo* imaging to aid co-registration, and landmarks including bifurcations, guide catheter location, and prominent calcium deposits used for localization. For each histopathological section, an experienced intravascular imaging investigator defined overall plaque classification, as well as measurements of lumen and EEL dimensions, fibrous cap thickness, lipid arc, and calcium arc.

**OCT image optimization to enhance plaque classification and plaque features**

We designed a novel artifact correction technique to identify and mitigate the effect of artifacts on tissue identification and measurements. After selecting the local-best frame subsection, the software matched the sub-image pixel intensity distributions to align pixel intensity profiles to reference sub-images. The matched images were then integrated into a single image which underwent noise reduction through a median filter to enhance image clarity. Adaptive filtering with binary masks derived from thresholding operations ensured preservation of essential features while mitigating the impact of outliers. This fully automated, parameter-free, and self-adaptive approach resulted in OCT images free of most common artifacts and with depth enhancement, making it easily extendable to diverse real-world OCT data.

**AutoOCT plaque definitions**

Low-risk lesions were defined as either normal vessel, adaptive intimal thickening (AIT), or pathological intimal thickening (PIT), with higher-risk lesions classified as thick-cap fibroatheroma (ThCFA), thin-cap fibroatheroma (TCFA), or fibrocalcific plaque. We based this definition of risk on pathological studies[41] and the landmark PROSPECT study[34] which showed Hazard Ratios (HRs) for ThCFA, fibrocalcific plaques, and TCFA were 1.09, 1.56 and 3.90 respectively. More detailed plaque classification was based on component measurements, defined according to histopathological description[41] and standard OCT definitions[6]. Where a parameter was not defined, a group consensus was taken to define the missing variable.

*Normal vessel*. Intima-media thickness <300μm and absence of lipid and absence of calcium.

*Adaptive intimal thickening (AIT)*. Intima-media thickness >300μm and lipid arc <90 degrees.

*Pathological intimal thickening (PIT)*. Intima-media thickness >500μm and lipid arc <90 degrees.

*Thick-cap fibroatheroma (ThCFA)*. Intima-media thickness >300µm, lipid arc >90 degrees, and FCT >75µm.

*Thin-cap fibroatheroma (TCFA)*. Intima-media thickness >300µm, lipid arc >90 degrees and FCT <75µm.

*Fibrocalcific Plaque.* Intima-media thickness >300µm, lipid arc <90 degrees, and calcium arc >30 degrees.

**Clinical validation studies**

*IBIS-4*

The Integrated Biomarker Imaging Study-4 OCT arm (IBIS-4, NCT00962416) assessed changes in OCT-defined plaque composition in non-culprit lesions in patients with STEMI undergoing PCI to the culprit lesion and receiving high-intensity statin treatment. Patient demographics and lesion characteristics are listed in the primary publication[7].

*CLIMA*

The CLIMA study (NCT02883088) studied patients undergoing OCT evaluation of the untreated proximal left anterior descending coronary artery in the context of clinically indicated coronary angiogram[5]. Patient characteristics are shown in **Table S4**.

# Supplemental Figures

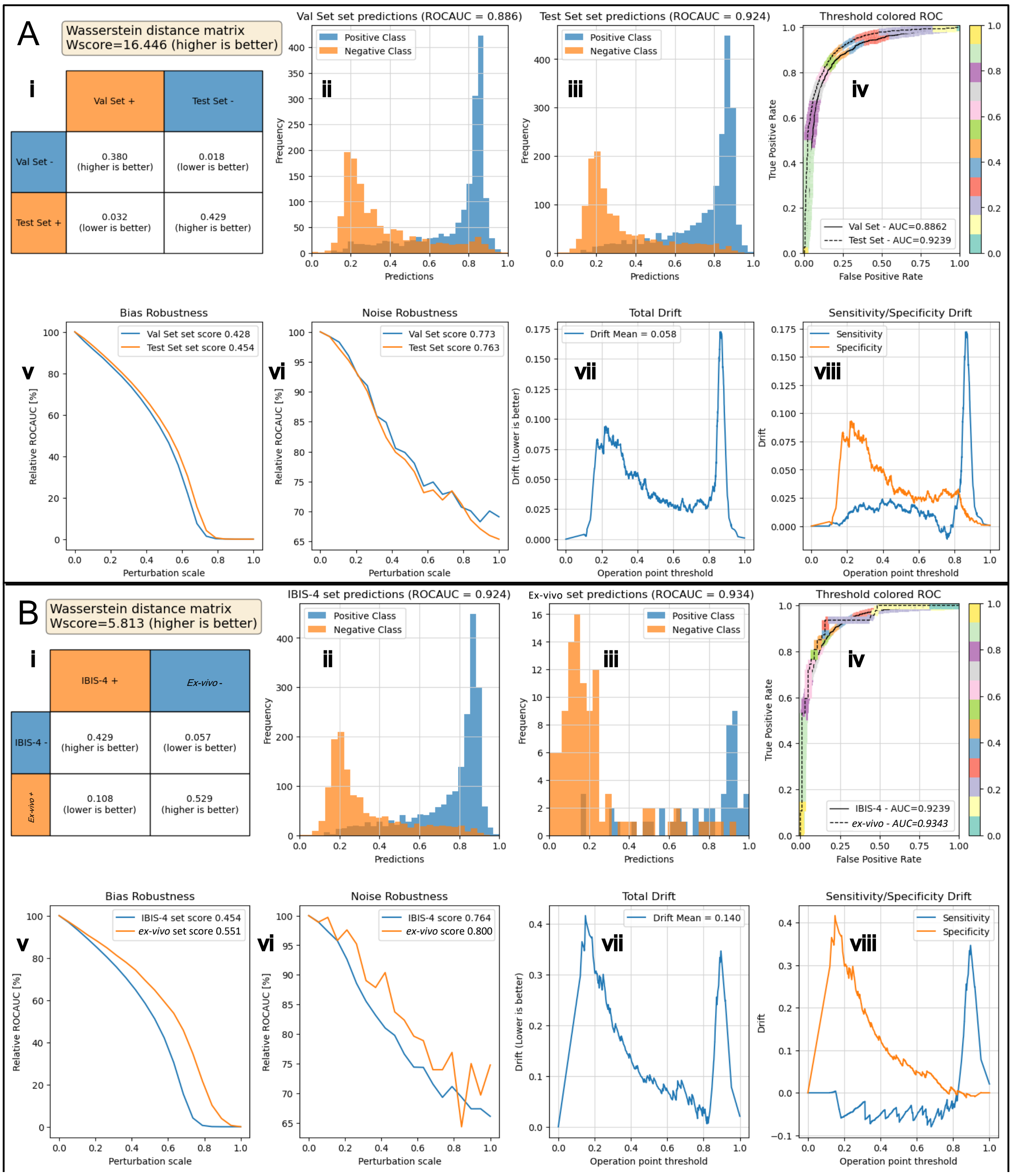


**Figure S1: Plaque classification consistency of model performance**
Assessing the consistency of model performance between **(A)** the internal validation and internal holdout data for IBIS-4 and **(B)** the internal IBIS-4 holdout and the external *ex-vivo* holdout data. As described in Roberts et. al.[33]; (i) The pairwise Wasserstein distances between the positive and negative classes of each dataset; (ii-iii) the histograms of the model outputs for the positive and negative samples for each dataset; (iv) a ROC curve for each dataset colored by operating point; (v-vi) robustness of the AUROC when model outputs are subject to differing levels of bias and Gaussian noise, respectively; (vii-viii) the total Euclidean distance between sensitivities and specificities at different operating points in aggregate and separated, respectively.

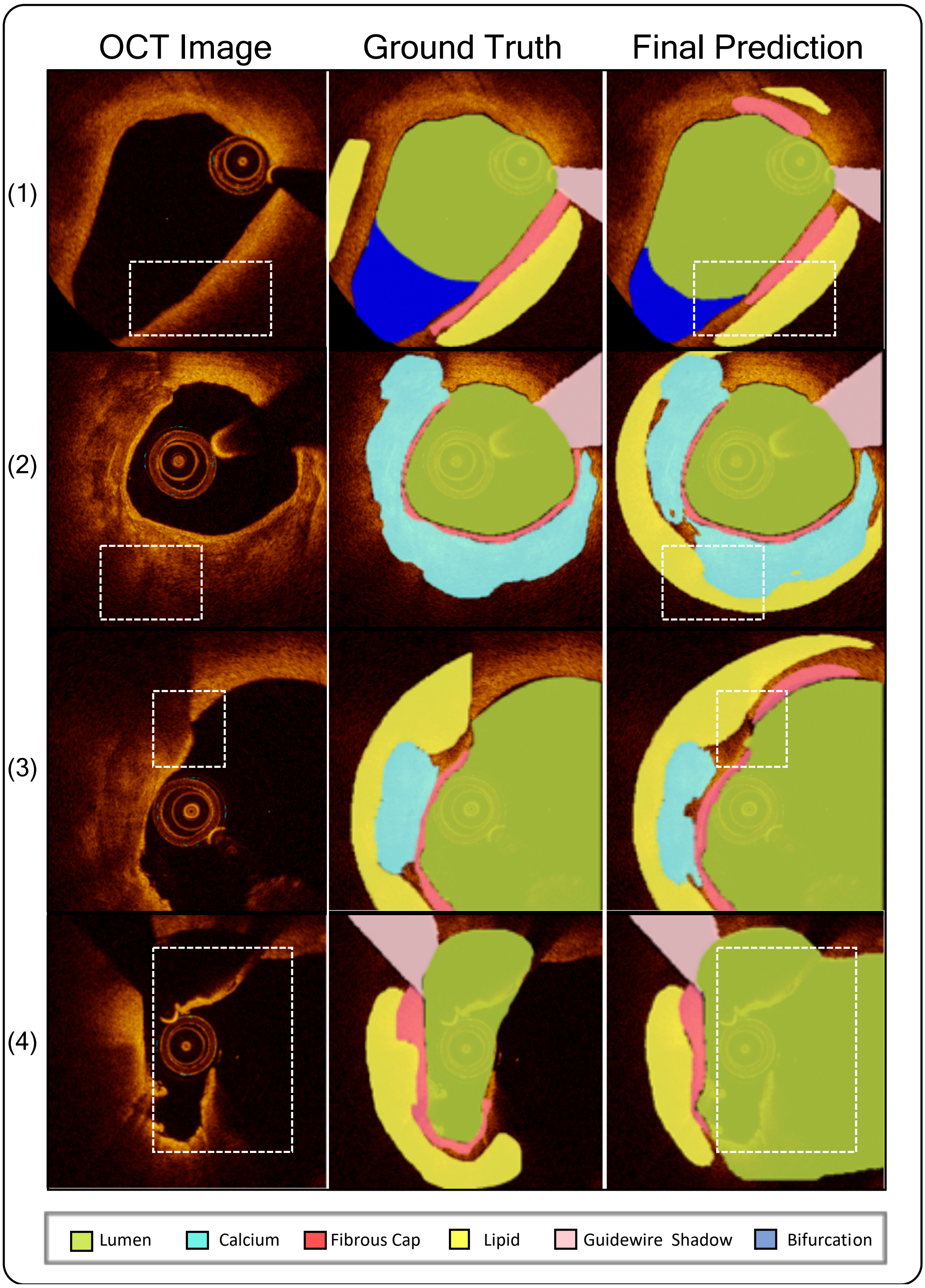


**Figure S2: Examples of AI and shared human-AI technical errors**
Examples of model segmentation in frames demonstrating OCT technical errors. From left to right, the raw OCT image, ground truth/manual annotations, and AutoOCT results after optimization are displayed, respectively. **(1)** large side branches cause lipid-like signal drop-out in vessel shoulder regions; **(2)** high calcium burden in fibrocalcific plaque causing lipid-like poor light penetration; **(3)** tangential signal drop-out leads to subjectivity in lipid and fibrous cap segmentation; **(4)** plaque rupture in a large vessel leads to errors in lumen segmentation. Outlined areas denote technical and segmentation errors.

# Supplemental Tables

| | Overall (n=14) | CV Death (n=8) | Non-CV Death (n=6) |
|---|---|---|---|
| Male, n (%) | 10 (71.4) | 8 (100.0) | 2 (33.3) |
| Age, y (SD) | 71.1 (11.8) | 76.8 (9.8) | 63.6 (10.3) |
| **Comorbidities, n (%)** | | | |
| Ischemic Heart Disease | 7 (50.0) | 6 (75.0) | 1 (16.7) |
| Cerebrovascular Disease | 2 (14.3) | 1 (12.5) | 1 (16.7) |
| Extra-cardiac Arteriopathy | 6 (42.9) | 4 (50.0) | 2 (33.3) |
| Diabetes Mellitus | 1(7.1) | 1 (12.5) | 0 (0.0) |
| Hypertension | 5 (35.7) | 3 (37.5) | 2 (33.3) |
| Cardiac Failure | 6 (42.9) | 4 (50.0) | 2 (33.3) |
| CTEPH | 3 (21.4) | 1 (12.5) | 2 (33.3) |

**Table S1: Demographics of post-mortem donors**
*CV indicates cardiovascular; CTEPH, chronic thromboembolic pulmonary hypertension.*

| | **Sensitivity (%)** | **Specificity (%)** | **Dice** |
|---|---|---|---|
| **Artery Component** | | | |
| Lumen | 0.99 (0.99 to 1.00) | 1.00 (0.99 to 1.00) | 0.99 (0.98 to 0.99) |
| Bifurcation | 0.89 (0.86 to 0.93) | 1.00 (0.99 to 1.00) | 0.89 (0.86 to 0.92) |
| EEL | 1.00 (0.99 to 1.00) | 0.99 (0.98 to 1.00) | 0.99 (0.98 to 0.99) |
| **Plaque Component** | | | |
| Lipid | 0.86 (0.80 to 0.93) | 1.00 (0.99 to 1.00) | 0.84 (0.77 to 0.92) |
| Calcium | 0.87 (0.80 to 0.94) | 1.00 (0.99 to 1.00) | 0.85 (0.77 to 0.92) |
| Fibrous Cap | 0.81 (0.68 to 0.94) | 1.00 (0.99 to 1.00) | 0.80 (0.68 to 0.91) |
| **Imaging Component** | | | |
| Guidewire Shadow | 0.98 (0.97 to 0.99) | 1.00 (0.99 to 1.00) | 0.96 (0.94 to 0.98) |
| | **Sensitivity (%)** | **Specificity (%)** | **Accuracy (%)** |
| **Miscellaneous Components** | | | |
| Guide Catheter | 0.97 (0.96 to 0.98) | 1.000 (1.00 to 1.00) | 0.98 (0.97 to 0.99) |
| Stent | 0.74 (0.71 to 0.78) | 0.99 (0.99 to 1.00) | 0.88 (0.87 to 0.89) |

**Table S2: Segmentation performance of AutoOCT on testing data set**
*EEL indicates external elastic lamina. Data expressed as percentage as appropriate. 95% CI are shown.*

| | Number of Patients | Number of Vessels | Baseline | Follow-up | Mean Change (95% CI) | P-value |
|---|---|---|---|---|---|---|
| **IBIS-4** | | | | | | |
| Region of Interest Length, mm | 83 | 153 | 27.71±10.53 | 27.63±10.54 | -0.07 (-0.45 to 0.31) | |
| Minimum Cap Thickness, µm | 27 | 31 | 64.88±19.89 | 87.88±38.08 | 24.41 (6.84 to 41.98) | 0.008 |
| Lipid arc, mean over frames, ° | 31 | 35 | 55.94±31.04 | 43.46±3.48 | -12.49 (-22.17 to -2.80) | 0.013 |
| **AutoOCT** | | | | | | |
| Region of Interest Length, mm | 83 | 153 | 27.66±10.55 | 27.59±10.57 | -0.07 (-0.44 to 0.30) | |
| Minimum Cap Thickness, µm | 27 | 31 | 62.86±28.35 | 81.80±33.41 | 18.93 (15.52 to 22.34) | <0.001 |
| Lipid arc, mean over frames, ° | 31 | 35 | 63.12±21.73 | 49.79±20.30 | -13.30 (15.17 to -11.51) | <0.001 |

**Table S3: Serial Vessel-Level OCT Analyses**
*Data presented are mean±SD.*

| | Patients with clinical event[a] | Patients without clinical event[a] | P-value |
|---|---|---|---|
| | (n = 31) | (n = 31) | |
| **Demographics** | | | |
| Age (yrs)[b] | 79 (63-84) | 67 (59-75) | 0.0581 |
| Male (%) | 20 (65%) | 27 (87%) | 0.0752 |
| **Cardiac risk factors** | | | |
| Left ventricle ejection fraction (%)[b] | 55 (45-60) | 53 (44-60) | 0.888 |
| Hypertension (%) | 24 (77%) | 17 (55%) | 0.107 |
| Smoking habit (%) | 12 (39%) | 7 (23%) | 0.271 |
| Family history of CAD (%) | 5 (16%) | 10 (32%) | 0.236 |
| Prior MI (%) | 6 (19%) | 4 (13%) | 0.730 |
| Prior PCI (%) | 8 (26%) | 4 (13%) | 0.335 |
| Diabetes mellitus (%) | 8 (26%) | 4 (13%) | 0.335 |
| CKD (GFR <60 mL/min/1.73 m2) (%) | 6 (19%) | 6 (19%) | 1.00 |
| Total cholesterol (mg/dL)[b] | 164 (154-206) | 171 (157-196) | 0.870 |
| LDL (mg/dL)[b] | 112 (91-144) | 103 (85-120) | 0.348 |
| HDL (mg/dL)[b] | 37 (31-51) | 44 (36-49) | 0.141 |
| Triglycerides (mg/dL)[b] | 116 (84-154) | 112 (85-133) | 0.666 |
| High-sensitivity C-reactive protein (mg/L)[b] | 6.75 (2.17-12.4) | 11.5 (3-25) | 0.181 |
| **Diagnosis** | | | |
| Stable angina (%) | 13 (45%) | 18 (58%) | 0.443 |
| Unstable angina (%) | 2 (7%) | 0 (0%) | 0.229 |
| NSTEMI (%) | 6 (21%) | 8 (26%) | 0.871 |
| STEMI (%) | 8 (28%) | 5 (16%) | 0.445 |

**Table S4: CLIMA patient characteristics**
*CAD, coronary artery disease; CKD, chronic kidney disease; GFR, glomerular filtration rate; HDL, high-density lipoprotein; LDL, low-density lipoprotein; MI, myocardial infarction; NSTEMI, non-ST-Elevation MI; PCI, percutaneous coronary intervention; STEMI, ST-elevation MI*

*a Composite of cardiac death and target LAD segment myocardial infarction.*
*b Expressed as median and interquartile range.*

| | All Population (n=62) | Patients with Clinical Events (n=31) | Patients without Clinical Events (n=31) | P-value |
|---|---|---|---|---|
| **Core Lab OCT findings** | | | | |
| Minimum lumen area <3.5 $mm^2$ (%) | 19 (30.6) | 12 (38.7) | 7 (22.6) | <0.001 |
| Fibrous cap thickness <75 µm (%) | 18 (29.0) | 13 (41.9) | 5 (16.1) | 0.004 |
| Maximum lipid arc >180° (%) | 26 (41.9) | 15 (48.4) | 11 (35.5) | <0.001 |
| **AutoOCT OCT findings** | | | | |
| Minimum lumen area <3.5 $mm^2$ (%) | 18 (29.0) | 12 (38.7) | 6 (19.4) | <0.001 |
| Fibrous cap thickness <75 µm (%) | 13 (21.0) | 9 (29.0) | 4 (12.9) | <0.001 |
| Maximum lipid arc >180° (%) | 30 (48.4) | 17 (54.8) | 13 (41.9) | <0.001 |

**Table S5: AutoOCT detection of features of plaque vulnerability**
*Clinical events defined as composite of cardiac death and target vessel myocardial infarction.*
*p-values given for reference.*

| | Sensitivity (%) | Specificity (%) | PPV (%) | NPV (%) |
|---|---|---|---|---|
| **Core Laboratory** | | | | |
| Minimum lumen area <3.5 $mm^2$ | 27.7 | 86.0 | 6.8 | 96.9 |
| Minimum fibrous cap thickness <75 µm | 40.6 | 83.9 | 8.6 | 97.4 |
| Maximum lipid arc extension >180° | 46.9 | 65.6 | 4.8 | 97.1 |
| **AutoOCT** | | | | |
| Minimum lumen area <3.5 $mm^2$ | 36.7 | 80.0 | 6.5 | 97.1 |
| Minimum fibrous cap thickness <75 µm | 30.0 | 86.7 | 7.9 | 97.0 |
| Maximum lipid arc extension >180° | 56.7 | 56.7 | 4.6 | 97.2 |

**Table S6: Accuracy of AutoOCT to detect higher-risk plaque features compared to core laboratory**
*NPV, negative predictive value; PPV, positive predictive value*
